# Healthcare Work and Organizational Interventions to Prevent Work-related Stress in Brindisi, Italy

**DOI:** 10.1016/j.shaw.2014.10.003

**Published:** 2014-10-18

**Authors:** Gabriele d'Ettorre, Mariarita Greco

**Affiliations:** 1 Local Health Authority, Brindisi, Health Unit of Occupational Prevention and Protection, Brindisi, Italy; 2 Local Health Authority, Brindisi, Department of Mental Health, Brindisi, Italy

**Keywords:** healthcare worker, stress assessment, work stress

## Abstract

**Background:**

Organizational changes that involve healthcare hospital departments and care services of health districts, and ongoing technological innovations and developments in society increasingly expose healthcare workers (HCWs) to work-related stress (WRS). Minimizing occupational exposure to stress requires effective risk stress assessment and management programs.

**Methods:**

The authors conducted an integrated analysis of stress sentinel indicators, an integrated analysis of objective stress factors of occupational context and content areas, and an integrated analysis between nurses and physicians of hospital departments and care services of health districts in accordance with a multidimensional validated tool developed in Italy by the National Network for the Prevention of Work-Related Psychosocial Disorders. The purpose of this retrospective observational study was to detect and analyze in different work settings the level of WRS resulting from organizational changes implemented by hospital healthcare departments and care services of health districts in a sample of their employees.

**Results:**

The findings of the study showed that hospital HCWs seemed to incur a medium level risk of WRS that was principally the result of work context factors. The implementation of improvement interventions focused on team development, safety training programs, and adopting an ethics code for HCWs, and it effectively and significantly reduced the level of WRS risk in the workplace.

**Conclusion:**

In this study HCW resulted to be exposed to occupational stress factors susceptible to reduction. Stress management programs aimed to improve work context factors associated with occupational stress are required to minimize the impact of WRS on workers.

## 1 Introduction

Healthcare organizations are continuously evolving models that are based on the effectiveness, efficiency, and appropriateness of health interventions. Ongoing technological innovations with developments in society and the current financial crisis results in the need to work with fewer staff and the consequent overwork increases pressure on healthcare workers (HCWs) to demonstrate changeability and resilience [1–3].

The organizational changes, which involve work time and intensity, type of employment contract, psychosocial factors at work, work–life balance, and health and safety policies within the organization, apparently interfere with employee health [4–8]. In addition, HCWs are intrinsically exposed to a variety of specific occupational stress factors in their work, which may cause discomfort and increase the likelihood of mistakes and practice errors [9]. A cause of discomfort is increased workload to ensure the achievement of higher clinical goals, which developing technologies increasingly allow. When employees perceive an increase in job demands, they are more likely to go on long-term sickness absence; by contrast, an increase in support at work lead employees to have fewer long spells of sickness absence [3,4].

Rather than interventions targeting individual behaviors, organizational-level workplace interventions are required to produce more sustainable effects on the health of healthcare employees. Based on the occupational health principle of “hierarchy of controls,” it is likely that interventions aimed at the level of the work organization or the work environment may produce more sustainable effects on the health of employees, compared to interventions focused primarily on individual-level characteristics. Furthermore, Montano et al [10–12] in a recent review emphasized that success rates are higher among more comprehensive interventions that simultaneously tackle material, organizational, and work-time related conditions. The purpose of this retrospective observational study was to detect and analyze (in different work settings) the level of work-related stress due to organizational change decisions in hospital healthcare departments and care services of health districts in a sample of their employees.

Based on the European Framework Agreement on Work-related Stress of October 8, 2004 [13], which was incorporated in Italy into Legislative Decree 81/08 and under which it is obligatory to make a valid and reliable evaluation of WRS, the authors conducted an integrated analysis of stress sentinel indicators and objective stress factors of occupational context and occupational content among hospital departments and among primary and community care services of health districts. The identification of these indicators could be useful in future work to identify the actions necessary to prevent WRS.

## 2 Materials and methods

The study was conducted in Brindisi, Italy from December 2011 to December 2013. To investigate the objective indicators of w.r.s, the authors conducted interviews with head physicians and head nurses of 114 hospital healthcare departments, and interviews with head physicians and head nurses of 98 primary and community care services of health districts.

The hospital healthcare departments of directly managed acute-care hospitals and rehabilitation hospitals provide hospital-based acute inpatient, outpatient, and rehabilitation care. These hospitals usually only provide secondary care. Health districts are geographical units responsible for coordinating and providing primary care and nonhospital-based specialist medicine. The interviews were conducted using a multidimensional validated tool developed by the Italian Network for the Prevention of Work-related Psychosocial Disorders in compliance with the Consultative Committee's specific requirements. This tool was tested on 800 companies listed by the Veneto Region ASL20 (regional NHS unit) Occupational Prevention, Hygiene, and Safety Service (Verona, Italy) and by the University of Verona (Verona, Italy) [14–17]. The tool identifies indicators of WRS risk in an organization under three headings: (1) sentinel events; (2) work content factors; and (3) work context factors (Table 1).

The study was performed as part of the obligatory evaluation of work-related stress, which is required by Italian Legislative Decree 81/08. This study required no formal approval by the local ethics committee.

The tool identifies three levels of risk: low (a score of 0–17), medium (a score 18–34), and high (a score >35) [16]. For each of the three areas of indicators, the tool identifies three levels of risk (Table 2). The actions needed depend on the level of risk and may vary from a monitoring plan for low risk to corrective measures and, if required, in-depth evaluation for medium and high risk. Through preparation by the authors, improvement plans oriented to solving critical organizational issues raised during the assessment were made specifically for each hospital department and health district; the plans were addressed to the participants and company's management. The authors have taken steps to train the participants regarding WRS. The necessary organizational corrective actions to prevent WRS were based on the results of the assessment. The training took place through lectures in two meetings with each participant about the organizational changes necessary to prevent WRS After implementing the improvement organizational actions, the authors assessed the level of stress of each hospital department and health district by interviewing the same head physicians and the head nurses. The participants were the same before and after the organizational interventions.

### 2.1 Statistical analysis

The statistical analysis of the data was based on the calculation of the average, the standard deviation, the distribution, and the range in accordance with the nature of individual variables. The differences between the means were compared using the Student test for continuous data. Differences were considered significant for values of *p* < 0.05.

## 3 Results

The results of our study on the occupational stress evaluation obtained by an objective approach and utilizing the multidimensional validated tool indicated that all hospital departments reported a medium level of WRS among physicians, compared to nurses. The validated tool indicated that all care services of the health districts showed a lower level of WRS among physicians than among nurses.

The mean values of the WRS index, as identified by the checklist before the implementation of improvement interventions, were significantly higher among physicians and nurses of hospital departments than among physician and nurses in care services of health district (Table 3). However, we found that the mean values of stress indicators were significantly higher among the physicians and nurses of hospital departments, compared with care services of health districts (Table 4). The analysis identified the objective stress factors associated with work and led to our suggestions for reducing the sources of WRS among HCWs in hospital departments and care services of health districts (Table 5).

After implementing improvement organizational interventions, the mean scores of the WRS index were significantly reduced for physicians [15.71 (after) vs. 22.16 (before); *p* < 0.05] and for nurses [16.14 (after) vs. 24.44 (before); *p* < 0.05] of hospital departments, and for physicians [9.81 (after) vs. 16.85 (before); *p* < 0.05] and for nurses [10.13 (after) vs. 17.00 (before); *p* < 0.05] of care services of health districts.

## 4 Discussion

The findings of our investigation indicate that hospital HCWs incur a greater risk of WRS compared with HCWs of care services of health districts. The analysis of work context area factors showed that all investigated hospital departments and care services of health districts were characterized by a medium risk level. Furthermore, the areas of sentinel events and work content factors evinced a low risk among hospital departments than among care services of health districts. Regarding context area factors, we did not find any statistically significant differences between physicians and nurses of hospital departments and physicians and nurses of care services of health districts (*p* > 0.05). By contrast, significant differences existed in the area of work content factors. In fact physicians and nurses of hospital departments showed a higher risk, compared with the same staff of care services of health districts (*p* < 0.05). These findings depend on the specificity of hospital healthcare activity that is characterized by typical stressors linked with hospital healthcare professions such as third-shift work, high exposure to physical and biological risks [18–25], variable and often nonprogrammable workloads, and work using technologies that require high responsibility [8,10]. These objective critical issues related to work content are apparently unsusceptible to modification because they are intrinsically characteristic of the hospital healthcare work and require organization of safety training courses among hospital HCWs to protect them.

The results of our investigation showed that work context was the priority area of organizational intervention to reduce WRS among hospital departments and care services of health districts. In this area, actions were focused on team development (i.e., working towards goals that include occupational safety, reflective dialogue and feedback among workers, supervisor support, feedback and involvement in decision making), on implementing safety training programs, and on adopting an ethics code for HCWs (Table 5).

Our study has several limitations. The analysis is imprinted on objective evaluation without consequent subjective analysis. However, some authors report that this type of evaluation is better than subjective analysis because it is not influenced by felt stress [12]. As a consequence of implementing corrective measures aimed at improving these aspects, the scores of objective WRS (as measured by the checklist) among the hospital departments and the care services of health districts were significantly reduced to a lower level of risk (*p* < 0.05) among hospital department HCWs than among care services HCWs of health districts HCW.

Job organization interventions aimed at improving the work context area appeared to configure as an instrument of primary prevention [3], and to improve workers' wellness with regard to preventing WRS [26–28]. In accordance with the results of this study, the Occupational Prevention and Safety Service of Local Health Authority proceeded to organize safety training programs to assist the workers in adopting effective safety strategies to manage occupational risks such as WRS and to ensure HCWs had better awareness of WRS risk.

## Conflicts of interest

All authors declare no financial or personal relationship with people or organizations that could inappropriately influence their work.

## Figures and Tables

**Table 1:** Indicators of work-related stress risk identified by the checklist∗

(I) Sentinel events (10 organizational indicators)	(II) Work content factors (4 indicators)	(III) Work context factors (6 indicators)
1. Work-related injuries	1. Work environment and work equipment	1. Function and organizational culture
2. Sick leave ∗	2. Task planning	2. Organizational role
3. Absences from work	3. Workload, work place	3. Career path
4. Unused vacations	4. Work schedule	4. Autonomy in decision making, job control
5. Job rotation		5. Interpersonal relationships at work
6. Turnover		6. Home/work interface, home/work balance
7. Disciplinary measures		
8. Requests for extra medical checks		
9. Work-related stress notifications		
10. Juridical petitions		

∗ More information can be found in “Work-elated stress risk assessment in Italy: a methodological proposal adapted to regulatory guidelines,” by B. Persechino et al., 2013, *Saf Health Work,* 4:95–9; “La valutazione dello stress lavoro-correlato: proposta metodologica,” by Network Nazionale per la Prevenzione Disagio Psicosociale nei Luoghi di Lavoro, 2010, *ISPESL* [In Italian]; and “Valutazione e gestione del rischio da stress lavoro correlate,” by INAIL, 2011.

**Table 2:** Risk levels identified by the scores of work-related stress indicators∗

Indicators	Low risk	Medium risk	High risk
Sentinel events	0–10†	11–20‡	21–30§
Work content factors	0–13	14–25	26–36
Work context factors	0–8	9–17	18–26

∗ More information can be found in “Valutazione e gestione del rischio da stress lavoro correlate,” by INAIL, 2011.

† Score converted into 0.

‡ Score converted into 2.

§ Score converted into 5.

**Table 3:** The mean values of the work-related stress index identified by the checklist before implementing improvement interventions

Healthcare unit	Level of WRS risk (mean ± SD)
Hospital departments (physicians)	22.16 ± 4.4∗
Hospital departments (nurses)	24.44 ± 4.6†
Care services of health districts (physicians)	16.85 ± 3.3
Care services of health districts (nurses)	17.00 ± 3.8

SD, standard deviation; WRS, work-related stress.

∗ Compared to physicians of health districts, *p* < 0.05.

† Compared to nurses of health districts, *p* < 0.05.

**Table 4:** The mean values of stress indicators between physicians and nurses

Healthcare unit	Sentinel events	Work content factors (mean ± SD)	Work context factors (mean ± SD)
Hospital department (physician)	0	12.20 ± 2.1∗	9.54 ± 1.8‡
Hospital department (nurses)	0	13.20 ± 1.4†	11.68 ± 2.1§
Care services of health districts (physicians)	0	6.80 ± 2.4	9.53 ± 1.7
Care services of health districts (nurses)	0	7.00 ± 2.9	9.57 ± 1.8

SD, standard deviation.

∗ Compared with physicians of health districts, *p* < 0.05.

† Compared with nurses of health districts, *p* < 0.05.

‡ Compared with physicians of health districts, *p* > 0.05.

§ Compared with nurses of health districts, *p* > 0.05.

**Table 5:** Work context critical issues and improvement interventions

Area of critical issues	Intervention
Function and organizational culture	Work towards goals that include occupational safety and wellness Adopt a safety management system Adopt a code of ethics for healthcare workers
Role within the occupational organization	Clear definition of occupational roles Knowledge of hierarchical roles for occupational safety Employee participation in corporate decisions
Relationship at work	Improve communication with management staff Improve reflective dialogue and feedback among workers
